# Prenatal alcohol exposure alters brain structure and neurocognitive outcomes for 6‐ to 7‐year‐old children in a South African birth cohort

**DOI:** 10.1111/acer.70048

**Published:** 2025-04-06

**Authors:** Chanellé J. Hendrikse, Shantanu H. Joshi, Jessica E. Ringshaw, Layla Bradford, Annerine Roos, Catherine J. Wedderburn, Nadia Hoffman, Tiffany Burd, Katherine L. Narr, Roger P. Woods, Heather J. Zar, Dan J. Stein, Kirsten A. Donald

**Affiliations:** ^1^ Division of Developmental Paediatrics, Department of Paediatrics and Child Health, Red Cross War Memorial Children's Hospital University of Cape Town Cape Town South Africa; ^2^ Department of Neurology, Ahmanson‐Lovelace Brain Mapping Center University of California Los Angeles California Los Angeles USA; ^3^ Department of Bioengineering University of California Los Angeles California Los Angeles USA; ^4^ Neuroscience Institute University of Cape Town Cape Town South Africa; ^5^ Department of Neuroimaging, Centre for Neuroimaging Sciences Kings College London London UK; ^6^ Department of Psychiatry and Mental Health University of Cape Town Cape Town South Africa; ^7^ Department of Paediatrics and Child Health, Red Cross War Memorial Children's Hospital University of Cape Town Cape Town South Africa; ^8^ Unit on Child and Adolescent Health, South African Medical Research Council (SAMRC) University of Cape Town Cape Town South Africa; ^9^ Department of Psychiatry and Biobehavioral Sciences University of California Los Angeles Los Angeles California USA; ^10^ The Semel Institute for Neuroscience and Human Behavior University of California Los Angeles Los Angeles California USA; ^11^ David Geffen School of Medicine University of California Los Angeles Los Angeles California USA; ^12^ South African Medical Research Council (SAMRC), Unit on Risk and Resilience in Mental Disorders University of Cape Town Cape Town South Africa

**Keywords:** early learning outcomes, neurodevelopment, prenatal alcohol exposure, structural magnetic resonance imaging

## Abstract

**Background:**

Several studies have demonstrated an association between prenatal alcohol exposure (PAE) and altered brain structure. However, more research is needed to understand how structural brain changes may influence neurocognitive performance in children with PAE at the age of school entry. We investigated the associations between PAE and cortical and subcortical gray matter morphology and whether PAE‐related structural brain changes mediate the associations between PAE and neurocognitive outcomes in 6‐ to 7‐year‐old children.

**Methods:**

One hundred fifty‐eight children (49 PAE, 109 unexposed controls; 46% female; mean age 76 ± 5 months) who participated in a brain imaging substudy of the population‐based Drakenstein Child Health Study were included. The children had moderate‐to‐high PAE without other substance exposure, except prenatal tobacco exposure. T1‐weighted brain structural scans were acquired using a 3T MRI scanner. General linear models and mediation analyses tested the associations of PAE with cortical and subcortical metrics and associated neurocognitive outcomes.

**Results:**

PAE was associated with a smaller total cortical surface area and had multivariate effects on regional cortical volume and surface area in the temporal lobe. The smaller volume and surface area of the left middle temporal gyrus mediated associations between PAE and neurocognitive outcomes for numeracy and mathematics and/or cognition and executive functioning. Findings persisted when adjusting for age, sex, maternal education, prenatal tobacco exposure, and, in volumetric and surface area models, intracranial volume.

**Conclusion:**

This study suggests that there is persistent altered brain structural development in children with PAE, consistent with previous findings in this cohort at infancy and age 2–3 years. Cortical changes in regions known to play a role in numeracy and semantic memory mediated associations between PAE and neurocognitive deficits, highlighting clinical relevance. Efforts to prevent PAE and improve neurocognitive development in children with PAE should be implemented as early as possible after birth.

## Graphical abstract

We investigated associations between prenatal alcohol exposure (PAE), cortical and subcortical morphology, and neurocognitive outcomes in 158 6‐ to 7‐year‐old children. PAE was associated with smaller total cortical surface area and regional cortical volume and surface area changes in the temporal lobe, adjusting for age, sex, maternal education, prenatal tobacco exposure, and intracranial volume. Morphological changes in the left middle temporal gyrus mediated associations between PAE and numeracy/mathematics and cognition/executive functioning. Exploratory analyses further revealed regional cortical morphological changes in other lobes.

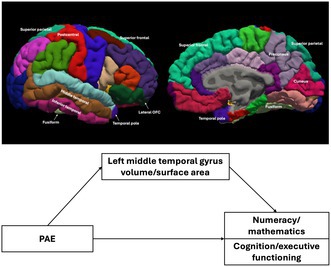

## INTRODUCTION

Maternal alcohol use during pregnancy is common globally despite growing awareness of the harmful health effects of prenatal alcohol exposure (PAE) on the developing fetus. In utero exposure to alcohol may cause fetal alcohol spectrum disorders (FASD), associated with a range of long‐term neurodevelopmental deficits spanning social, emotional, behavioral, and cognitive domains. PAE may also be associated with physical disabilities, abnormal facial features, or organ defects (Kar et al., 2021; Mattson et al., 2019).

The negative effects of PAE may stem from alcohol‐induced brain changes in the developing fetus and child (Mattson et al., 2019; Moore & Xia, 2022). In typically developing, unexposed children, cortical morphological changes in volume, surface area, and thickness across the early years of life are thought to underlie age‐related development in cognitive abilities (Cox et al., 2018). Cortical maturational processes begin before birth (Bethlehem et al., 2022; Storsve et al., 2014) and are, therefore, highly susceptible to toxic in utero exposures. For example, PAE may alter the functioning of the GABAergic system, which is crucial for neuronal survival, differentiation, and connectivity (Ikonomidou et al., 2000; Marguet et al., 2022). Dysfunction of this system may therefore result in altered brain volumes and synaptic density in children with PAE (Alhowail, 2022; Licheri & Brigman, 2021). The impact of PAE on these neurobiological processes during embryonic and fetal development may persist in influencing trajectories of cortical morphological development long after birth.

This is supported by neuroimaging studies indicating that moderate to severe PAE can alter cortical and subcortical developmental trajectories throughout childhood and adolescence (Donald et al., 2015; Hendrickson et al., 2018; Lebel et al., 2011; Moore & Xia, 2022). PAE‐related brain structural changes in several cortical and subcortical regions have been widely reported (Donald et al., 2015). Widespread PAE‐related alterations in cortical volume, thickness, or surface area across frontal, temporal, parietal, and occipital lobes (Chen et al., 2012; Robertson et al., 2016; Sowell et al., 2008; Subramoney et al., 2022; Treit et al., 2014, 2017; Zhou et al., 2011), and volumetric reductions of subcortical regions, such as the hippocampus and thalamus, have been reported (Chen et al., 2012; Nardelli et al., 2011; Zhou, Rasmussen, et al., 2018a). Some studies show smaller global brain volumes or atypical brain growth over time (Lebel et al., 2012). Despite some inconsistencies in findings from cross‐sectional imaging investigations (Rajaprakash et al., 2014; Subramoney et al., 2022), emerging evidence suggests that cortical morphological changes following PAE may underlie poorer verbal learning, spatial memory performance, neuropsychological functioning, and intelligence scores in individuals with PAE (Chu et al., 2022; Mattson et al., 2019).

Inconsistencies in the literature may be due to several factors. Firstly, given that different cortical features and regions have heterogeneous developmental trajectories (Bethlehem et al., 2022; Lyall et al., 2015), the specific neurodevelopmental footprint of PAE might depend on the child's age as well as the region or metric under investigation (Rajaprakash et al., 2014). Secondly, the teratogenic impact of PAE may be influenced by the timing and severity of exposure (Lees et al., 2020; Petrelli et al., 2018), the socio‐demographic characteristics of the mother and child, exposure to additional toxins in utero, and the postnatal environment. Lower maternal educational attainment and income, poorer nutrition, and pre‐ and postnatal tobacco exposure may augment the neurodevelopmental impact of PAE and associate with more severe FASD symptomatology (Xia et al., 2023). Several studies have also shown that brain structural changes in PAE may differ between males and females (Inkelis et al., 2020; Nardelli et al., 2011; Subramoney et al., 2022; Treit et al., 2017). It is crucial to adjust for the influence of these factors in studies on the effects of PAE on brain structure.

We have conducted a longitudinal brain imaging study nested within the Drakenstein Child Health Study (DCHS; Zar et al., 2015) to investigate the neurodevelopmental effects of prenatal and early‐life exposures in South African children from birth to school age (Donald et al., 2018; Stein et al., 2015; Wedderburn et al., 2020). In this cohort, PAE was associated with altered patterns of white matter microstructural development across the first 6 years of life (Donald et al., 2023; Roos et al., 2021), lower total gray matter volume at the neonatal stage and at 2–3 years old (Donald et al., 2016; Subramoney et al., 2022), and regional volumetric and/or surface area changes in basal ganglia (specifically the putamen) and parietal lobe regions at 2–3 years old (Subramoney et al., 2022). Although no differences in cortical thickness between 2‐ and 3‐year‐old children with PAE and unexposed controls were found (Subramoney et al., 2022), PAE‐related cortical thickness abnormalities may manifest later, as suggested by other studies in older children and adolescents (Robertson et al., 2016; Sowell et al., 2002, 2008; Zhou et al., 2011; Zhou, Rasmussen, et al., 2018a).

Here, we investigated the associations of PAE with subcortical volume and cortical volume, surface area, and thickness and neurocognitive outcomes in 6‐ to 7‐year‐old children from the DCHS. Additionally, we investigated the potential mediating role of PAE‐related brain structural changes in the relationship between PAE and neurocognitive outcomes contributing to school readiness. Given that a considerable portion of the children were scanned previously in the DCHS, we had the rare opportunity to build upon earlier findings in this cohort.

## MATERIALS AND METHODS

### Study setting and participants

This study included 6‐ to 7‐year‐old children participating in the brain imaging substudy of the population‐based DCHS between August 2018 and December 2022. The DCHS is located in a low‐income, peri‐urban district with a population of about 200,000 people, approximately 60 km outside of Cape Town, South Africa. Details on this community have been described previously (Donald et al., 2018; Stein et al., 2015; Zar et al., 2015).

The children's mothers (aged 18 years or older) were recruited during pregnancy (between 20 and 28 weeks gestation) as part of the DCHS between March 2012 and March 2015 while attending routine antenatal care at one of two primary healthcare clinics in the Drakenstein area. The mothers were followed through childbirth, and a subset of mothers was approached about having their children participate in the brain imaging substudy. At the neonatal age, the children were selected for the brain imaging substudy to represent the overall study population risk profile, which included PAE (Wedderburn et al., 2020). They did not have any of the following exclusion criteria: (1) medical comorbidity, (2) low Apgar score (<7 at 5 min), (3) neonatal intensive care admission, (4) maternal use of illicit drugs during pregnancy, and (5) child HIV infection.

For the current time point (age 6–7 years), children who were active in the cohort, staying within the study area, and who were scanned at previous time points were prioritized for neuroimaging. The children in the final analysis sample for this study (*n* = 158) were representative of the full DCHS cohort at birth (*n* = 1143) in terms of sex, maternal education, prenatal tobacco exposure, and maternal antenatal and postnatal depression. Ultimately, this sample (*n* = 158) was over‐represented for maternal HIV infection (31% of the current sample) compared with participants from the full cohort (*n* = 1143; 22% with maternal HIV). Nevertheless, the PAE and control groups of the current sample were balanced in terms of maternal HIV exposure, and sensitivity analyses with maternal HIV exposure as an additional covariate did not alter the significance of our main findings.

**FIGURE 1:**
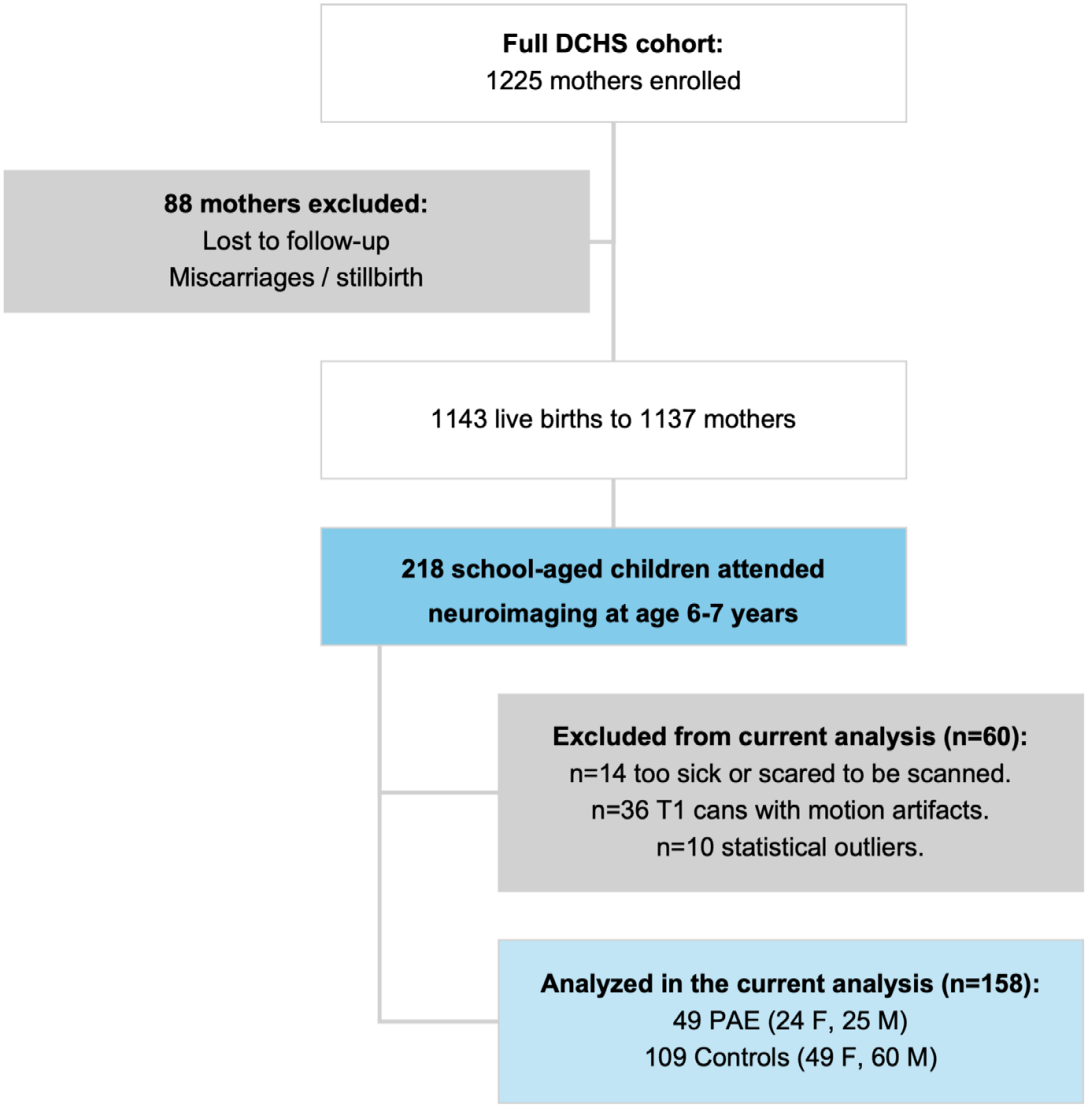
Flowchart of participation in the Drakenstein Child Health Study (DCHS).

Mothers provided written informed consent, and children provided assent to participate. A total of 218 children attended a neuroimaging visit at age 6–7 years, of which 158 children had usable scan segmentation output. Differences between the children with usable scan segmentation output and those without usable scan output are described in the results. The children also completed a neurodevelopmental assessment at this age. See Figure 1 for an outline of participation in the DCHS.

### Measures

#### Socio‐demographics and child anthropometrics

Maternal and child socio‐demographic and health data were collected via self‐report, physical examinations, and biological specimens (e.g., urine samples for toxicology and cotinine screening, blood samples for HIV testing). Child anthropometric measurements (i.e., weight, height, and head circumference) were recorded at the brain imaging visit.

#### Prenatal alcohol and tobacco exposure

Mothers prospectively completed the Alcohol, Smoking, and Substance Involvement Screening Test (ASSIST; Humeniuk et al., 2008) during pregnancy, and retrospectively reported alcohol use during pregnancy at the neonatal brain imaging visit. The ASSIST is a widely validated World Health Organization (WHO) scale (Donald et al., 2016; Humeniuk et al., 2008). Children of mothers with an antenatal ASSIST score greater than 11 for the alcohol questions, as recommended by the WHO, or who reported drinking two or more units of alcohol per occasion, two or more times per week during any trimester, were included in the PAE group. The remaining children were classified as no or low exposure controls. Maternal smoking status was also obtained from the ASSIST, and active tobacco smoking during pregnancy was determined using urine cotinine tests. Mothers who reported illicit substance use on the ASSIST were excluded from participation.

#### Neurocognitive outcomes

Children completed the early learning outcomes measure (ELOM), a standardized instrument developed to assess early learning program outcomes and school readiness in South African children between 4 and 6 years old. It comprises 23 items and five subscales: (1) gross motor development, (2) fine motor coordination and visual motor integration, (3) emergent numeracy and mathematics, (4) cognition and executive functioning, and (5) emergent literacy and language. Respectively, these subscales measure (1) large muscle control, (2) small muscle use and visual motor integration, (3) understanding of numerical concepts, space, symbols, shapes, and sizes, (4) working memory, impulse control, problem‐solving skills, critical thinking, and ability to form concepts, and (5) language use and communication skills. Studies have shown that the ELOM is reliable and internally consistent in assessing early learning outcomes in children of diverse socio‐economic status and ethnolinguistic backgrounds (Anderson et al., 2021; Dawes et al., 2020; Snelling et al., 2019). Concurrent validity of the ELOM total and subscale scores with the widely used Wechsler Preschool and Primary Scale of Intelligence Fourth Edition (WPPSI‐IV) full scale composite and corresponding indices scores has been demonstrated (Anderson et al., 2021).

#### Maternal depression

The parent study measured maternal depression as part of its psychosocial battery. The Beck Depression Inventory (BDI) and Edinburgh Postnatal Depression Scale (EPDS) were used to measure maternal depressive symptomatology during pregnancy and 6 months after giving birth. These scales are widely validated (Beck et al., 1988; Cox et al., 1987). Total scores for the BDI and EPDS were calculated as the sum of scores for all items of each scale (BDI = 21 items; EPDS = 10 items). A score >19 on the BDI or >12 on the EPDS was used to indicate the presence of depression. Considering that maternal depression could influence child brain outcomes and neurodevelopment (Roos et al., 2022), we examined whether rates of maternal depression were different between groups to determine whether it needed to be included as a covariate in this sample.

### Image acquisition

High‐resolution T1‐weighted brain structural scans were acquired on a 3T Siemens Skyra MRI scanner (Erlangen, Germany) situated at the Cape Universities Brain Imaging Centre (CUBIC), Groote Schuur Hospital, Cape Town. Imaging parameters were as follows: TR = 2500 ms; TE = 3.35 ms; flip‐angle = 8 degrees; slice thickness = 1.0 mm; 176 slices; voxel size: 1.0 × 1.0 × 1.0 mm; and acquisition time = 7 min 12 s. The children were awake during scanning. A trained research assistant or parent was present with them in the scanner suite during the scan (Wedderburn et al., 2020).

### Image processing and quality control

All scans were inspected for completeness and motion or other artifacts before processing. Automated cortical reconstruction and subcortical segmentation were performed using FreeSurfer version 7.1.1 implemented on the Centre for High Performance Computing (CHPC) Rosebank, Cape Town, Sun Intel Lengau cluster. FreeSurfer has been reliably used in prior studies to study the effects of PAE in pediatric samples (Biffen et al., 2020). Details of the FreeSurfer reconstruction pipeline are described on the developer webpage. FreeSurfer extracts the volume of the total cerebral cortex, total gray matter, total white matter, and seven subcortical regions (Fischl et al., 2002), as well as the volume, thickness, and surface area of 34 cortical regions according to the Desikan–Killiany atlas (Desikan et al., 2006).

The cortical parcellation output for all participants was visually inspected for accuracy and quality by two independent reviewers according to the ENIGMA cortical quality control protocol (https://enigma.ini.usc.edu/protocols/imaging‐protocols/). Scans with excessive motion artifacts or insufficient overall segmentation quality were excluded (*n =* 36). Scans with extreme values (±3 times the interquartile range from the 1st or 3rd quartiles) for any of the cortical or subcortical ROIs were also excluded (*n* = 10).

### Statistical analysis

Statistical analysis was performed using SPSS version 28 with significance set at *p* < 0.05. Group differences (i.e., PAE vs. control) for maternal and child socio‐demographics, anthropometrics, and psychosocial risk variables were calculated using independent‐samples *t*‐tests, Pearson chi‐squared tests, or Fischer's exact tests. Thereafter, we assessed (1) PAE associations with global and regional brain structural metrics, (2) PAE associations with neurocognitive outcomes, and (3) the mediating role of PAE‐related brain structural changes in the association between PAE and neurocognitive outcomes.

#### PAE associations with global and regional brain structural metrics

Univariate analysis of variance (ANOVA) models were run to investigate associations of PAE with (1) total gray matter volume, (2) total cortex volume, (3) total subcortical gray matter volume, (4) total cortical surface area, and (5) mean overall cortical thickness. Multivariate analysis of variance (MANOVA) models were used to examine associations of PAE with regional (6) subcortical volume and regional cortical (7) volume, (8) surface area, and (9) thickness for each of the four lobes and cingulate cortex separately (see Table S1 for a complete list of ROIs). While prior studies have often included subjects across a wide age range and reported findings from all cortical lobes (Rockhold et al., 2023), we explored cortical and subcortical gray matter metrics across the brain to ensure that no meaningful PAE‐related effects and neurodevelopmental associations in this specific age group were missed. Significant multivariate main effects for groups of ROIs were followed up by post hoc univariate ANOVAs for each ROI. The false discovery rate (FDR) method (Benjamini & Hochberg, 1995) was applied to correct for multiple comparisons for ROIs grouped by lobe. Uncorrected results were, however, also reported, and exploratory univariate analyses with all ROIs were conducted to avoid Type II errors. Partial eta‐squared values were reported to indicate effect size.

All models were run adjusting for child sex, age, maternal education, and prenatal tobacco exposure (henceforth referred to as fully adjusted models). These factors are known to be associated with child brain development (Lyall et al., 2015; Uban et al., 2017; Zhu et al., 2023). To account for natural inter‐individual variation in overall head/brain size independently of PAE, we included intracranial volume (ICV) as an additional covariate in volumetric and surface area models (i.e., models 1–4 and 6–8 as above) as these metrics scale with head/brain size (Backhausen et al., 2021). This approach also allowed us to measure the relative effects of PAE on specific brain regions (Hyatt et al., 2020; Wedderburn et al., 2020), which may otherwise be confounded by overall brain volume reductions in exposed children (Inkelis et al., 2020).

The models were also run without adjusting for prenatal tobacco exposure, with no covariates, and while adjusting for ICV only (volumetric and surface area models). These findings may be useful for demonstrating the relevance of the chosen covariates and are reported in Table S1.

#### PAE associations with neurocognitive outcomes

Group differences in the total and subscale scores of the ELOM were examined with fully adjusted models using separate univariate ANOVAs.

#### Mediation analyses

**FIGURE 2:**
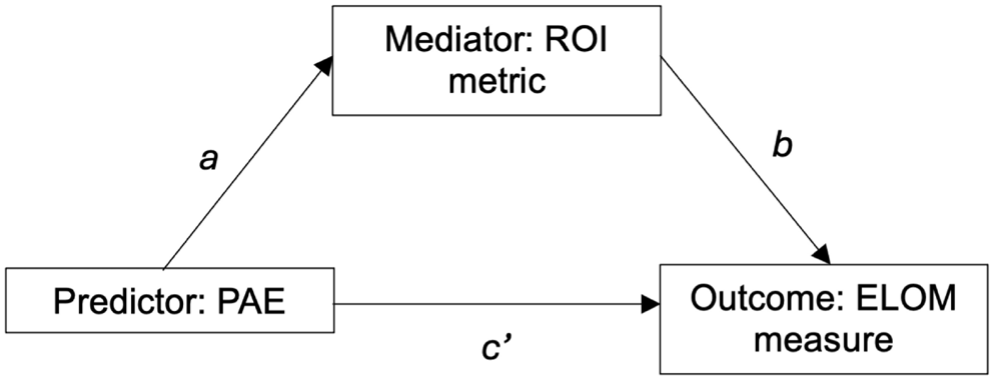
Illustration of the simple mediation model.

Mediation analyses with PAE as the predictor were run to test whether brain structure mediated significant associations between PAE and neurocognitive outcomes (Figure 2). A bootstrapping method was applied using the SPSS Process Macro (Hayes, 2022). The mediation model first tests the direct associations *a, b*, and *c′* (Figure 2). The symbol *c′* denotes the relationship between the predictor and the outcome in the presence of the mediator. Thereafter, it assesses the product of the associations *a* and *b* (i.e., a*b) to determine the indirect effect, that is, whether the mediator (ROI metric) mediates the relationship between the predictor (PAE) and the outcome (ELOM measure). Mediation is considered to be present if the indirect effect is significant, that is, the 95% bootstrap confidence interval does not include zero (Hayes, 2022). Mediation models were run while adjusting for child age, sex, maternal education, prenatal tobacco exposure, and—in volumetric and surface area models—total intracranial volume.

## RESULTS

### Descriptives

Of the 218 children who attended scanning, 158 children had usable segmentation output. There were no significant socio‐demographic differences between participants with usable MRI data (*n* = 158) and those with unusable MRI data (*n* = 60) in terms of age, sex, maternal education, PAE status, and prenatal tobacco exposure (Table S2). However, participants with usable MRI data had higher ELOM total scale scores (*F*(1) = 8.164, *p* = 0.005), as well as higher scores for the ELOM fine motor coordination (*F*(1) = 6.034, *p* = 0.015), emergent numeracy and mathematics (*F*(1) = 11.289, *p* = 0.001), and cognition and executive functioning (*F*(1) = 4.139, *p* = 0.044) subscales, adjusting for age, sex, maternal education, prenatal tobacco exposure, and PAE. This suggests that children with better neurocognitive functioning were more likely to have usable MRI scans, potentially because they were less likely to move during scanning.

Table 1 summarizes the sample (*n* = 158: 49 PAE, 109 controls) socio‐demographic, anthropometric, and psychosocial risk characteristics. There were no group differences in sex, household size, or maternal characteristics, such as age, educational attainment, employment status, total household income, marital status, HIV infection, and antenatal depression at enrollment, or postnatal depression 6 months after giving birth.

**TABLE 1:** Sample characteristics.

Total sample (*n* = 158)			
Variable^a^	PAE group (*n* = 49)	Control group (*n* = 109)	*p*
Child socio‐demographics			
Age, months	74 ± 5 (69–88)	76 ± 5 (69–90)	0.001
Sex, boys	25 (51)	60 (55)	0.639
Gestation, weeks	38 ± 3 (30–42)	39 ± 2 (31–42)	0.021
Child anthropometrics			
Height, cm^b^	113 ± 6 (100–126)	117 ± 6 (105–128)	<0.001
Weight, kg	19 ± 2 (14–25)	21 ± 4 (16–33)	<0.001
Head circumference, cm	51 ± 2 (48–59)	52 ± 2 (45–57)	<0.001
Mother socio‐demographics at enrolment			
Age, years	27 ± 6 (18–41)	28 ± 6 (18–42)	0.506
Educational attainment			
Primary	6 (12)	5 (5)	0.058
Some secondary	30 (61)	53 (49)	
Completed secondary	12 (25)	46 (42)	
Any tertiary	1 (2)	5 (5)	
Employed	13 (27)	36 (33)	0.414
Monthly income^c^			
<ZAR 1000	40 (82)	82 (77)	0.669
ZAR 1000–ZAR 5000	9 (18)	21 (20)	
>ZAR 5000	0 (0)	3 (3)	
Married/cohabitating	16 (33)	51 (47)	0.096
Household size	5 ± 2 (1–10)	5 ± 3 (1–13)	0.141
Maternal psychosocial risk factors			
Tobacco use during pregnancy	34 (69)	20 (18)	<0.001
HIV infection	11 (23)	38 (35)	0.119
Antenatal depression: above threshold^d^	14 (29)	21 (23)	0.391
Postnatal depression 6 months postpartum: above threshold^e^	5 (16)	8 (13)	0.651

Abbreviations: HIV, human immunodeficiency virus; PAE, prenatal alcohol exposure; ZAR, South African rand.

^a^ Values for continuous variables are presented as: mean ± standard deviation (range). Values for categorical variables are presented as: number (%).

^b^ Missing height data for *n* = 1 control participant.

^c^ Missing income data for *n* = 3 control participants.

^d^ Missing Beck Depression Inventory (BDI) data (*n* = 17: 1 PAE, 16 controls).

^e^ Missing Edinburgh Postnatal Depression Scale (EPDS) data (*n* = 64: 18 PAE, 46 controls).

Although statistically significant, group differences in child age at scanning (*t*(156) = −3.247, *p* = 0.001) and gestational age at delivery (*t*(156) = −2.336, *p* = 0.021) were small. The PAE group was, on average, 2 months younger at scanning and 1 week less at birth than the control group. This age difference may have contributed to lower average anthropometric measurements among the PAE compared with the control group at scanning (height, weight, and head circumference were all *p* < 0.001). Moreover, as expected, there was a higher prevalence of maternal active tobacco smoking during pregnancy in the PAE compared with the control group (*X* ^2^(1) = 39.143, *p* < 0.001).

### PAE effects on global brain metrics

Table 2 reports the associations of PAE with global brain metrics. PAE had a significant main effect on total cortical surface area (*F*(1) = 4.125, *p* = 0.044), which was smaller in the PAE group (*M* = 166,931 mm^2^, SD = 15,248 mm^2^) compared with the unexposed group (*M* = 175,930 mm^2^, SD = 15,595 mm^2^), this finding did not survive FDR correction (corrected *p* = 0.120).

**TABLE 2:** Uncorrected PAE effects on global brain metrics^a^.

Metric	Direction of effect in PAE	*F* (df)	*p*	Partial eta^2^
Total cortex volume	ns.	3.561 (1)	0.061	0.023
Total gray matter volume	ns.	3.279 (1)	0.072	0.021
Total subcortical gray matter volume	ns.	0.040 (1)	0.842	0.000
Total cortical surface area	↓	4.125 (1)	0.044	0.027
Mean overall cortical thickness	ns.	0.329 (1)	0.567	0.002

Abbreviations: ns, not significant; PAE, prenatal alcohol exposure.

^a^ Covariates: intracranial volume (in volumetric and surface area models only), child age, sex, maternal education, prenatal tobacco exposure.

### PAE effects on regional cortical metrics

**FIGURE 3:**
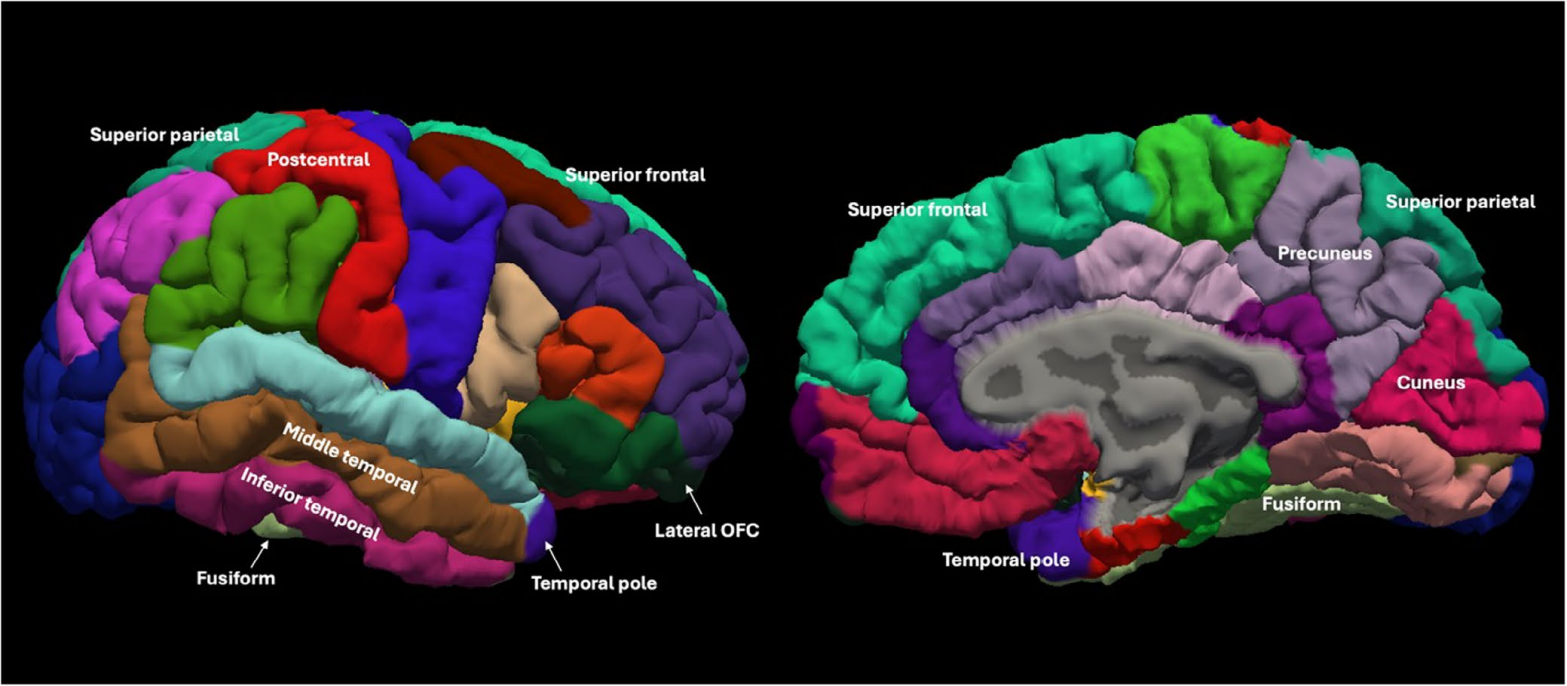
Cortical regions of the Desikan–Killiany atlas (Desikan et al., 2006) with significant univariate prenatal alcohol exposure (PAE) effects in the main (Table 3; Section PAE effects on regional cortical metrics) and exploratory analyses (Table S1; Section Exploratory univariate PAE effects on individual ROIs). All effects are illustrated on the right hemisphere.

PAE had significant main effects on regional cortical volume (CV) and regional cortical surface area (CSA) of the temporal lobe (CV: *p* = 0.024; CSA: *p* = 0.001). The effect for regional CSA survived FDR correction (corrected *p* = 0.005), whereas the effect for CV did not (corrected *p =* 0.120). Post hoc univariate ANOVAs revealed lower CV and CSA of the left middle temporal gyrus (CV: *p* = 0.030; CSA: *p* = 0.010) and the right fusiform gyrus (CV: *p* = 0.027; CSA: *p* = 0.044), as well as lower CSA of the right inferior temporal gyrus (*p* = 0.025) and the left temporal pole (*p* = 0.049) in the PAE compared with the control group. None of these univariate findings survived FDR correction. The results are reported in Table 3 and illustrated in Figure 3. PAE had no significant main effects on regional CV, CSA, or CT of the other cortical lobes or cingulate cortex.

**TABLE 3:** PAE effects on regional cortical volume and surface area in the temporal lobe.

Metric/Region	L/R	Direction and/or size of effect in PAE	*F* (df)	*p*		Partial eta^2^
				Uncorrected	FDR corrected	
Volume						
Multivariate main effect		Large	1.816 (18)	0.024	0.120	0.200
Univariate effects						
Middle temporal gyrus	L	↓ Small	4.821 (1)	0.030	0.270	0.031
Fusiform gyrus	R	↓ Small	5.016 (1)	0.027	0.270	0.032
Surface area						
Multivariate main effect		Large	2.529 (18)	0.001	0.005*	0.254
Univariate effects						
Middle temporal gyrus	L	↓ Small	6.835 (1)	0.010	0.180	0.043
Fusiform gyrus	R	↓ Small	4.130 (1)	0.044	0.212	0.027
Inferior temporal gyrus	R	↓ Small	5.099 (1)	0.025	0.212	0.033
Temporal pole	L	↓ Small	3.949 (1)	0.049	0.212	0.025

*Note*: Covariates: intracranial volume (in volumetric and surface area models only), child age, sex, maternal education, and prenatal tobacco exposure.

Abbreviations: PAE, prenatal alcohol exposure; ns, not significant.

* Survived FDR correction.

### Exploratory univariate PAE effects on individual ROIs

Exploratory univariate ANOVAs for individual ROIs in the other cortical lobes revealed lower CSA of the left superior frontal (*p* = 0.048) and left lateral orbitofrontal (*p* = 0.028) cortices, as well as lower CV of the left (*p* = 0.007) and right (*p* = 0.021) lateral orbitofrontal cortices in the frontal lobe. In the parietal lobe, lower CV and CSA of the right superior parietal (CV: *p* = 0.038; CSA: *p* = 0.008) and right precuneus (CV: *p* = 0.016; CSA: *p* = 0.006) cortices were found. PAE Effects on CSA of the parietal ROIs survived FDR correction (adjusted *p* = 0.040 for both regions). In terms of cortical thickness (CT), exploratory univariate ANOVAs revealed increased thickness of the left cuneus cortex (*p* = 0.008; occipital lobe) and right postcentral gyrus (*p* = 0.014; parietal lobe) in children with PAE compared with controls; however, these effects did not survive FDR correction (adjusted *p* = 0.064 and 0.140, respectively). These results are reported in full in Table S1. Figure 3 illustrates the anatomical locations of these regions on the Desikan–Killiany atlas (Desikan et al., 2006).

### PAE effects on subcortical volume

PAE had no effects on total or regional subcortical gray matter volumes at this age (see Table S1).

### PAE associations with neurocognitive outcomes

Table 4 reports the full results of the associations between PAE and the total and subscale scores of the ELOM. PAE was associated with lower total scale scores for the ELOM (*F*(1) = 10.192, *p* = 0.002), as well as poorer performance on the emergent numeracy and mathematics (*F*(1) = 12.849, *p* < 0.001), cognition and executive functioning (*F*(1) = 4.899, *p* = 0.029), and emergent language and literacy subscales (*F*(1) = 6.501, *p* = 0.012).

**TABLE 4:** PAE associations with ELOM total and subscale scores^a^.

Measure/domain	Means and standard deviations		Direction of effect in PAE^b^	Univariate effects^b^		
	PAE group (*n* = 37)	Control group (*n* = 82)		*F* (df)	*p*	Partial eta^2^
Total scale score	61.50 ± 11.835	69.29 ± 11.426	↓	10.192 (1)	0.002	0.083
Gross motor development	11.39 ± 4.171	12.60 ± 4.508	ns.	0.784 (1)	0.378	0.007
Fine motor coordination and visual motor integration	16.95 ± 3.243	17.49 ± 2.328	ns.	0.987 (1)	0.323	0.009
Emergent numeracy and mathematics	11.99 ± 3.367	14.31 ± 3.474	↓	12.849 (1)	<0.001	0.102
Cognition and executive functioning	9.54 ± 4.198	11.59 ± 3.463	↓	4.899 (1)	0.029	0.042
Emergent language and literacy	11.62 ± 4.265	13.30 ± 3.745	↓	6.501 (1)	0.012	0.054

Abbreviations: ELOM, early learning outcomes measure; ns, not significant; PAE, prenatal alcohol exposure; SD, standard deviation.

^a^ The sample size for analyses with the ELOM was smaller (*n* = 119: 37 PAE, 82 CON). Due to COVID‐19 restrictions on in‐person meetings, 39 participants had missing ELOM data during ongoing data collection.

^b^ Model adjusting for child age, sex, maternal education, and prenatal tobacco exposure.

### Brain associations with neurocognitive outcomes

Table 5 summarizes the significant associations between learning outcome measures and brain structural metrics of ROIs associated with PAE (see Table S3 for results in full).

**TABLE 5:** Summary of brain structural metrics associations with ELOM total and subscale scores^a^.

Metric/Region	L/R	ELOM measure (*p‐*values)^a^			
		Total score	Num. & Math.	Cogn. & Exec.	Lang. & Lit.
Volume^b^					
Middle temporal	L	ns.	*r* (112) = 0.275, *p* = 0.003	ns.	ns.
Fusiform	R	ns.	*r* (112) = 0.266, *p* = 0.004	ns.	ns.
Surface area^b^					
Middle temporal	L	*r* (112) = 0.237, *p* = 0.011	*r* (112) = 0.289, *p* = 0.002	*r* (112) = 0.234, *p* = 0.012	ns.

Abbreviations: PAE, prenatal alcohol exposure; ELOM, early learning outcomes measure; ns., not significant.

^a^ Missing data: 12 PAE, 27 controls. Total sample *n* = 119.

^b^ Covariates: child age, sex, maternal education, prenatal tobacco exposure, and intracranial volume.

### Mediation analysis

**FIGURE 4:**
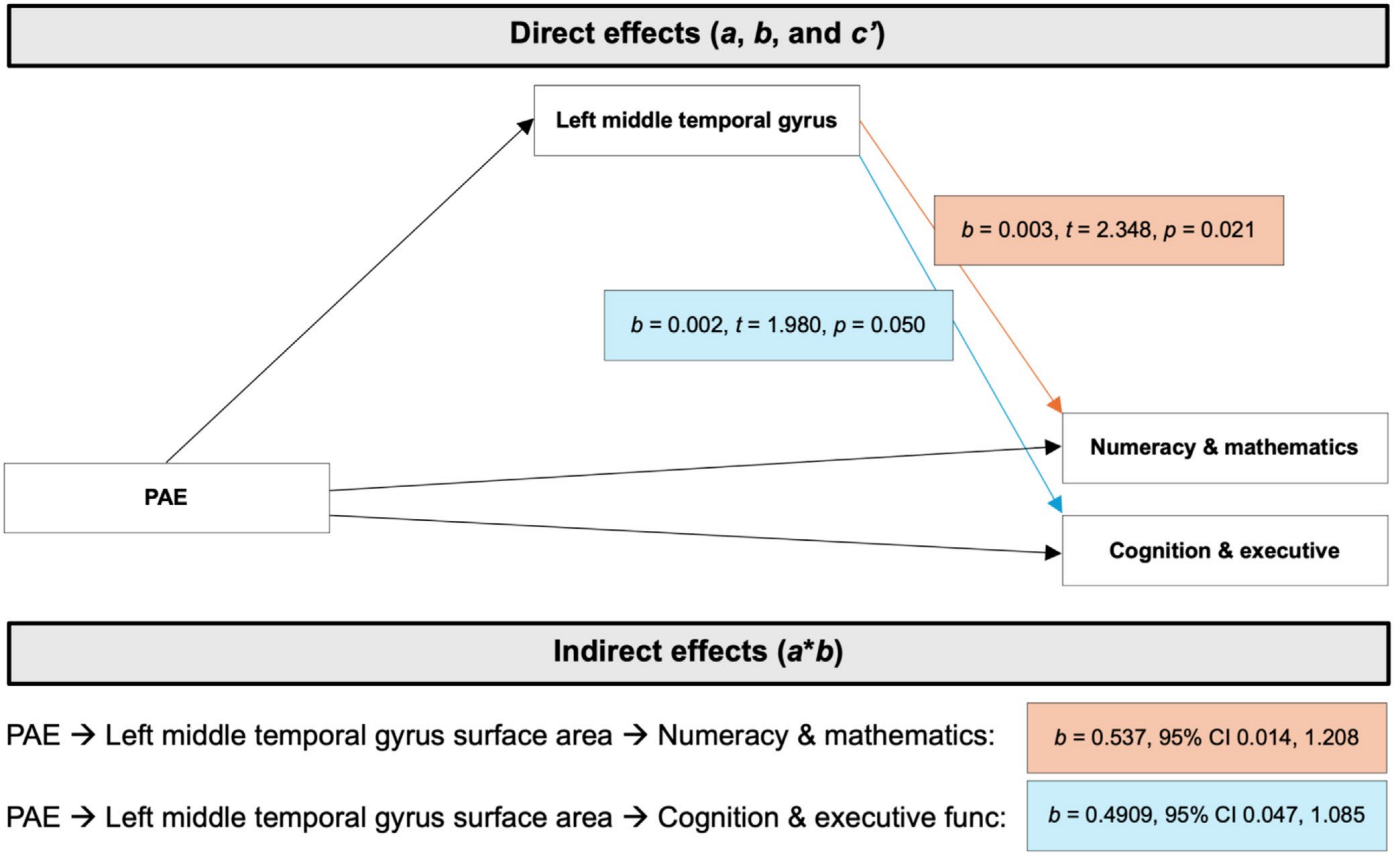
Illustration of the mediating role of surface area of the left middle temporal gyrus in the associations between prenatal alcohol exposure (PAE) and the early learning outcomes measure (ELOM) numeracy and mathematics (results indicated in orange rectangles) and cognition and executive functioning (results indicated in blue rectangles) subscales.

We conducted mediation analyses, in which PAE was independently associated with ROI metrics and learning outcome measures. The direct and indirect (via cortical morphology) associations of PAE with ELOM outcomes are reported in Table 6 and Figure 4.

**TABLE 6:** Mediation analysis results for the ELOM.

Metric/Region	L/R	ELOM	Fully adjusted associations (*N* = 119)^a^, ^b^					Mediation
			PAE – ROI	ROI–ELOM meas.	PAE–ELOM meas. (direct effect)	PAE–ROI–ELOM meas. (indirect effect)		
			*p*	*p*	*p*	95% CI: Lower limit	95% CI: Upper limit	
Volume								
Middle temporal	L	Num.	0.018	0.019	0.003	0.197	1.064	Yes
Fusiform	R	Num.	0.044	0.019	0.002	−0.005	0.914	No
Surface area								
Middle temporal	L	Total	0.002	0.009	0.078	−0.164	3.366	No
		Num.	0.002	0.021	0.005	0.014	1.208	Yes
		Cogn.	0.002	0.050	0.099	0.047	1.085	Yes

Abbreviations: CI, confidence interval; Cogn., cognition and executive functioning subscale; ELOM, early learning outcomes measure; L/R, left or right hemisphere; Lang., language and literacy subscale; Num., numeracy and mathematics subscale; PAE, prenatal alcohol exposure; ROI, region of interest.

^a^ Missing data: 12 PAE, 27 controls. Total sample *n* = 119.

^b^ Model adjusting for child age, sex, maternal education, prenatal tobacco exposure, and intracranial volume.

Mediation results were found for the left middle temporal gyrus. Specifically, cortical volume and surface area of the left middle temporal gyrus, respectively, mediated the associations between PAE and ELOM scores for the numeracy and mathematics subscale (volume: 95% CI: 0.197, 1.064; surface area: 95% CI: 0.014, 1.208). Surface area of the left middle temporal gyrus also mediated the relationship between PAE and ELOM scores for the cognition and executive functioning subscale (95% CI: 0.047, 1.085).

## DISCUSSION

We examined PAE associations with cortical and subcortical gray matter morphology in 6‐ to 7‐year‐old children from a low‐to‐middle‐income setting in South Africa. Children with PAE had global and regional cortical gray matter alterations and poorer neurocognitive performance compared with their unexposed peers. PAE‐related cortical morphological changes of the left middle temporal gyrus mediated neurocognitive outcomes. To our knowledge, this study is one of the first to demonstrate the potential mediating role of this region in the association between PAE and neurocognitive outcomes in school‐aged children.

### PAE effects on brain structure

PAE was associated with smaller total cortical surface area; however, this effect did not survive FDR correction. Nevertheless, a significant multivariate effect of PAE on regional cortical surface area in the temporal lobe was found; this effect was large, survived FDR correction, and was supported by a similar effect in terms of volume. Post hoc univariate analyses revealed smaller volume and/or surface area of several specific temporal lobe cortical regions, including the left middle temporal, right inferior temporal, and right fusiform gyri and the left temporal pole. While none of these effects survived FDR correction, they correspond with other studies with similarly aged and older children or adults that have also demonstrated PAE effects on volume or surface area of temporal lobe regions (Sowell et al., 2002).

Moreover, our findings of smaller total and regional cortical surface area in the PAE group are consistent with prior volumetric, surface area or gyrification findings at earlier ages in this (Donald et al., 2016; Subramoney et al., 2022) and other cohorts (Hendrickson et al., 2018; Infante et al., 2015). In this cohort at the age of 2–4 weeks (Donald et al., 2016), PAE was associated with lower total gray matter volume, while in a follow‐up analysis at age 2–3 years, PAE was associated with lower total cortical surface area and volume. When adjusting for similar covariates as in the present analysis, PAE was associated with decreased volume and/or surface area of several parietal lobe regions, including the bilateral precuneus cortex, in 2–3 year‐old children (Subramoney et al., 2022). In exploratory analyses, we similarly observed lower volume and surface area of the right precuneus cortex in the current age group. This suggests that the earlier findings in this cohort demonstrating emerging patterns of altered parietal lobe volumetric and gyral development following PAE may persist to school age.

From a neurodevelopmental perspective, an increase in cortical surface area is considered to reflect gyral development (i.e., *gyrification*, simply described as the folding patterns on the surface of the brain; Rakic, 2009). Cortical surface area expansion allows for more compact neural wiring, which may augment efficient neural processing (Kinney & Volpe, 2018). Conversely, reduced cortical folding and synaptic density may be linked to less efficient neural processing. Our findings of smaller CSA in children with PAE may, therefore, reflect less mature or protracted gyral development across the first 6 years of life. This, in turn, may manifest as a range of neurocognitive deficits often observed in children with PAE. Indeed, reduced cortical gyrification has been linked with lower intelligence quotients in children with PAE (Hendrickson et al., 2018). Similarly, in the current study, reduced volume and/or surface area of temporal lobe regions were associated with poorer performance in important neurocognitive functions at school‐entry age, including numeracy/mathematics and cognition/executive functioning.

Exploratory analyses further revealed PAE effects on cortical thickness. Children with PAE showed reduced thickness of the right postcentral gyrus (parietal lobe) and left cuneus cortex (occipital lobe). However, these effects should be interpreted with caution given that main multivariate effects for cortical thickness in these lobes were not present. Furthermore, interpreting cortical thickness findings is challenging due to inconsistencies in the literature. While some studies report no cortical thickness associations in toddlers with PAE (Subramoney et al., 2022), others have reported directionally and regionally different findings in older children and adolescents. Inconsistencies in the literature may be due to the sensitivity of cortical thickness measurements to image acquisition factors, including scanner hardware, sequence parameters, analysis software, and motion artifacts (Walhovd et al., 2016), as well as the fact that many prior studies have included subjects spanning broad age ranges (Sowell et al., 2008; Zhou et al., 2011; Zhou, Rasmussen, et al., 2018a). Nevertheless, a small longitudinal study (*n* = 11 FASD, *n* = 21 controls) with children between the ages of 6 and 15 years found altered patterns of cortical thinning among children with FASD (Treit et al., 2014). One other study in 10‐ to 11‐year‐old South African children found an association between PAE and increased cortical thickness of voxel clusters located in the right occipital cortex (including the cuneus and pericalcarine cortex) and right parietal lobe (including the supramarginal/postcentral gyrus; Robertson et al., 2016). Increased cortical thickness has been linked with poorer neurocognitive functioning in typically developing children (Sowell et al., 2008) and smaller palpebral fissure length in children with PAE (Sowell et al., 2008). Replication of these findings in larger samples and longitudinal investigations are needed to get a clearer picture of the potential impact of PAE on regional cortical thickness.

### Poorer neurocognitive outcomes in PAE

PAE was associated with significantly poorer performance in learning outcome domains, including numeracy/mathematics and cognition/executive functioning. This finding corresponds with several other studies demonstrating suboptimal school readiness and/or poorer academic performance in children with PAE (Lebel et al., 2012; Mattson et al., 2001, 2019), which may, in turn, negatively affect long‐term self‐esteem and social and emotional development (Gibbard et al., 2003).

### Mediating role of cortical morphology in the relationship between PAE and neurocognition

A major finding of this study was that smaller volume and/or surface area of the left middle temporal gyrus mediated the association(s) between PAE and neurocognitive outcomes related to numeracy/mathematics and/or cognition/executive functioning. The left middle temporal gyrus, together with several other ROIs that showed significant or trend associations with PAE in exploratory analyses—including the fusiform, inferior parietal, and parahippocampal gyri and parts of the prefrontal cortex—has been shown to be involved in semantic memory processing (Binder et al., 2009). Semantic memory processing is important for understanding, storing, and retrieving conceptual knowledge about the world, including people, objects, actions, relations, self, and culture, acquired through experience (Binder et al., 2009). The middle temporal gyrus has also been shown to be important for integrating prior knowledge with an ongoing current dialogue (Raykov et al., 2022) as well as mathematical problem‐solving (Wang et al., 2022; Zhou, Li, et al., 2018b). It is therefore plausible that cortical morphology of this region was associated with the cognition/executive functioning and numeracy/mathematics subscales of the ELOM in the present study.

### Strengths, limitations, and future recommendations

This study has several strengths and limitations. Firstly, since this group had been imaged before, we could refer to the findings of our earlier studies at different time points to improve our understanding of when the observed cortical morphological changes might have started to appear. While recent years have witnessed an increase in prospective, follow‐up studies of the neurodevelopmental effects of PAE (Hendrickson et al., 2018; Lebel et al., 2012; Treit et al., 2014), evidence on the impact of PAE on distinct trajectories of brain development is still emerging. A limitation of this study is that we were not able to examine the longer‐term impact of PAE on cortical morphological development and associated neurocognitive outcomes. There is a need for expanding longitudinal work with research using large samples and well‐matched control groups. Regular scans should be done throughout infancy, childhood, *and* adolescence, to better understand whether these PAE‐related cortical changes represent the start or continuation of neurodevelopmental trajectories that may become more evident at a later age.

Another limitation is that more children with PAE had prenatal tobacco exposure than the children without PAE. However, this was an expected finding (Uban et al., 2023) and excluding children with concurrent prenatal tobacco exposure would have resulted in a much smaller and potentially unrepresentative sample. Moreover, we were not able to assess the effects of timing and duration of alcohol exposure, the nutritional adequacy of the children's diets, or preschool environments. Previous studies have shown an impact of these factors on brain development, and this would be interesting to examine in future work (Autti‐Rämö et al., 2002; Rajaprakash et al., 2014; Robertson et al., 2016). Nevertheless, maternal drinking during pregnancy was measured prospectively, which is a strength of this study considering that the vast majority of past studies on the brain structural effects of PAE have been retrospective in nature (Nguyen et al., 2017).

An interesting observation emerging from this study was that the participants with usable MRI data from the parent study on average had better neurocognitive functioning compared with participants with unusable (or artifactual) MRI data, which did not meet quality checking standards due to motion or other artifacts. This may reflect that participants with usable MRI data were more cooperative and less likely to move during scanning. Furthermore, this may have implications for our analysis in the sense that children at the lower end of neurocognitive functioning in the parent sample may have been underrepresented in our analysis.

## CONCLUSION

This study suggests delayed or inadequate milestone development in children with PAE that is underpinned by PAE‐related cortical changes. Consistent with the literature, our findings suggest that the specific neurocognitive functions affected by PAE may depend on the extent and location of cortical gray matter changes as well as the child's age. Our findings highlight the critical need to expand preventive strategies for PAE, as well as the clinical relevance of early detection through comprehensive assessment. Targeted interventions and support services for children affected by PAE need to be more widely implemented in an effort to improve neurodevelopmental and neurocognitive outcomes and school readiness. The findings further deepen current understanding of the potential longer‐term PAE effects on brain structural development and highlight potential avenues for future longitudinal investigations.

## AUTHOR CONTRIBUTIONS

**Chanellé J. Hendrikse:** Conceptualization; methodology; formal analysis; software; data curation; visualization; writing—original draft. **Shantanu H. Joshi, Katherine L. Narr, and Roger P. Woods:** Conceptualization; methodology; writing—review and editing. **Jessica E. Ringshaw, Layla Bradford, Annerine Roos, and Catherine J. Wedderburn:** Investigation; data curation; writing—review and editing. **Nadia Hoffman and Tiffany Burd:** Project administration; data curation; writing—review and editing. **Heather J. Zar:** Conceptualization; methodology; supervision; funding acquisition; writing—review and editing. **Dan J. Stein:** Conceptualization; methodology; supervision; writing—review and editing. **Kirsten A. Donald:** Conceptualization; methodology; investigation; supervision; project administration; funding acquisition; writing—review and editing.

## FUNDING INFORMATION

This work was supported by the Bill and Melinda Gates Foundation (grant number: OPP 1017641), National Institute on Alcohol Abuse and Alcoholism (grant numbers: R21AA023887, R01AA026834–01), US Brain and Behavior Research Foundation (grant number: 24467), Collaborative Initiative on Fetal Alcohol Spectrum Disorders (CIFASD) (grant number: U24 AA014811), South African Medical Research Council, UK Government's Newton Fund (grant number: NAF002/1001), Wellcome Trust (grant number: 203525/Z/16/Z), South Africa's National Research Foundation (grant numbers: 105865 and 120432), ABMRF/The Foundation for Alcohol Research, and Harry Crossley Foundation. The content is solely the responsibility of the authors and does not necessarily represent the official views of the funders.

## CONFLICT OF INTEREST STATEMENT

The authors declare that they have no known competing financial interests or personal relationships that could have appeared to influence the work reported in this paper.

## Supporting information


Table S1

Table S2

Table S3

## Data Availability

Study data are available from the corresponding author upon reasonable request.
