# Supplementary material for: Prenatal alcohol exposure alters brain structure and neurocognitive outcomes for 6‐ to 7‐year‐old children in a South African birth cohort
Source: Alcohol Clin Exp Res (Hoboken). 2025 Apr 6;49(5):1028–41. doi: 10.1111/acer.70048 (PMC12098808; doi:10.1111/acer.70048)
Supplement: Supplementary file 2 — Table S2 [file ACER-49-1028-s002.docx]

**Supplementary Table 2: Brain structural metrics associations with ELOM total and subscale scores.**

|  |  | **ELOM measure (p-values)^a^** | | | | | |
| --- | --- | --- | --- | --- | --- | --- | --- |
| **Metric/Region** | **L/R** | **Total score** | Gross mot. | Fine mot. | Num. & Math. | Cogn. & Exec. | Lang. & Lit. |
| *Volume^b^* |  |  |  |  |  |  |  |
| Lateral orbitofrontal | L | 0.291 | 0.270 | 0.556 | 0.499 | 0.872 | 0.427 |
|  | R | 0.441 | 0.461 | 0.846 | 0.854 | 0.825 | 0.320 |
| Superior parietal | R | 0.050 | 0.339 | 0.266 | 0.218 | 0.075 | 0.205 |
| Precuneus | R | 0.293 | 0.566 | 0.893 | 0.908 | 0.281 | 0.168 |
| Middle temporal | L | 0.095 | 0.989 | 0.892 | 0.003 | 0.113 | 0.445 |
| Fusiform | R | 0.104 | 0.614 | 0.429 | 0.004 | 0.287 | 0.818 |
| *Surface area^b^* |  |  |  |  |  |  |  |
| Lateral orbitofrontal | L | 0.117 | 0.217 | 0.450 | 0.571 | 0.376 | 0.140 |
| Superior frontal | L | 0.009 | 0.649 | 0.257 | 0.009 | 0.113 | 0.007 |
| Superior parietal | R | 0.012 | 0.183 | 0.068 | 0.110 | 0.018 | 0.255 |
| Precuneus | L | 0.161 | 0.573 | 0.487 | 0.966 | 0.072 | 0.163 |
| Middle temporal | L | 0.011 | 0.970 | 0.077 | 0.002 | 0.012 | 0.214 |
| Fusiform | R | 0.071 | 0.965 | 0.183 | 0.052 | 0.074 | 0.287 |
| Inferior temporal | R | 0.406 | 0.676 | 0.311 | 0.438 | 0.300 | 0.755 |
| Temporal pole | L | 0.753 | 0.695 | 0.288 | 0.358 | 0.576 | 0.502 |
| *Thickness^c^* |  |  |  |  |  |  |  |
| Postcentral | R | 0.047 | 0.578 | 0.047 | 0.056 | 0.017 | 0.942 |
| Cuneus | L | 0.002 | 0.165 | 0.136 | 0.017 | 0.006 | 0.066 |

Abbreviations: PAE, prenatal alcohol exposure.

^a^Missing data: 12 PAE, 27 controls. Total sample *n* = 119.

^b^Model adjusting for child age, sex, maternal education, prenatal tobacco exposure, and intracranial volume.

^c^Model adjusting for child age, sex, maternal education, and prenatal tobacco exposure.
