# Supplementary material for: Prenatal alcohol exposure alters brain structure and neurocognitive outcomes for 6‐ to 7‐year‐old children in a South African birth cohort
Source: Alcohol Clin Exp Res (Hoboken). 2025 Apr 6;49(5):1028–41. doi: 10.1111/acer.70048 (PMC12098808; doi:10.1111/acer.70048)
Supplement: Supplementary file 3 — Table S3 [file ACER-49-1028-s001.docx]

**Supplementary Table 3. Comparison of participants with usable versus unusable MRI data.**

|  | **Usable MRI data**  **(*n* = 158)** | **Unusable MRI data**  **(*n* = 60)** | **Statistics** |
| --- | --- | --- | --- |
| **Age, months** | 76 ± 5 | 75 ± 4 | *t* (216) = -1.737, *p* = 0.085 |
| **PAE status** | | | *X^2^* (1) = 0.021, *p* = 0.885 |
| PAE | 49 (31%) | 18 (30%) |  |
| Control | 109 (69%) | 42 (70%) |  |
| **Sex** | | | *X^2^* (1) = 1.093, *p* = 0.296 |
| Female | 73 (46%) | 23 (38%) |  |
| Male | 85 (54%) | 37 (62%) |  |
| **Maternal education** | | | *X^2^* (3) = 5.540, *p* = 0.136 |
| Primary | 11 (7%) | 0 (0%) |  |
| Some secondary | 83 (53%) | 36 (60%) |  |
| Secondary | 58 (37%) | 20 (33%) |  |
| Any tertiary | 6 (4%) | 4 (7%) |  |
| **Prenatal tobacco exposure** | | | *X^2^* (1) = 0.329, *p* = 0.566 |
| Yes | 54 (34%) | 23 (38%) |  |
| No | 104 (66%) | 37 (62%) |  |
| **Maternal HIV infection** | | | *X^2^* (1) = 0.392, *p* = 0.531 |
| Yes | 49 (31%) | 16 (27%) |  |
| No | 109 (69%) | 44 (73%) |  |
| **Early Learning Outcomes Measure (ELOM) mean scores^a^** | | | |
| Total scale | 67 ± 12 | 60 ± 12 | *F* (1) = 8.164, *p* = 0.005 |
| Gross motor dev. | 12 ± 4 | 11 ± 5 | *F* (1) = 1.513, *p* = 0.221 |
| Fine motor coord. | 17 ± 3 | 16 ± 3 | *F* (1) = 6.034, *p* = 0.015 |
| Numeracy & math. | 14 ± 4 | 11 ± 3 | *F* (1) = 11.289, *p* = 0.001 |
| Cogn. & exec. | 11 ± 4 | 9 ± 4 | *F* (1) = 4.139, *p* = 0.044 |
| Language & lit. | 13 ± 4 | 12 ± 4 | *F* (1) = 0.407, *p* = 0.525 |

^a^Data available for 149 participants (119 with usable MRI data and 30 with unusable MRI data).
